# Fifty Shades of Immune Defense

**DOI:** 10.1371/journal.ppat.1003110

**Published:** 2013-02-07

**Authors:** Michael F. Criscitiello, Paul de Figueiredo

**Affiliations:** 1 Comparative Immunogenetics Laboratory, Texas A&M University, College Station, Texas, United States of America; 2 Department of Veterinary Pathobiology, Texas A&M University, College Station, Texas, United States of America; 3 Borlaug Center, Texas A&M University, College Station, Texas, United States of America; 4 Department of Microbial and Molecular Pathogenesis, Texas A&M Health Science Center, College Station, Texas, United States of America; 5 Department of Plant Pathology & Microbiology, College Station, Texas, United States of America; Duke University Medical Center, United States of America

## Overview

In their struggle to survive and thrive, all living things must defend themselves from predatory attack. Microbes, in the form of parasites, bacteria, fungi, and viruses, are life's most accomplished predators. Therefore, all living things have evolved mechanisms to defend against them. Historically, biological defense systems have been classified into two broad categories—innate systems that provide nonspecific defense against invading pathogens and adaptive systems that provide long-lasting defense against attack by specific pathogens. Recently, a growing body of literature in comparative immunology has indicated that these categories may not be as distinct as was originally believed. Instead, a variety of immune mechanisms that share properties of both innate and adaptive systems have been recently elucidated. Here, we summarize five key facts about the newly appreciated shades of grey between innate and adaptive defense systems.

## (1) Innate and Adaptive Immunity Are No Longer Black and White; There Are Increasing Shades of Grey

The innate immune system has conventionally been viewed as a relatively simple set of molecules and processes that defends cells and organisms against invading pathogens. Innate immune systems use chemical, biochemical, or mechanical barriers to prevent pathogen attack. These systems, however, do not confer specific protection to organisms against pathogens that have assaulted them in the past; that is, classical innate systems do not provide immunological memory. Recently, the boundary between innate and adaptive systems has become blurred by an emerging appreciation of the many shades of immunological memory. In humans and other jawed vertebrates, which provide the best studied example of immunological memory, clonally expanded populations of antigen-specific lymphocytes mediate the memory responses and confer long-term protection against re-infection. Adoptive transfer experiments in which lymphocytes from immunized animals were transferred to naïve congenic siblings have elegantly demonstrated that a persistent population of specialized memory cells is the mechanism by which immunological memory is conferred. Importantly, the concepts that emerged from these kinds of experiments formed the foundation of our thinking about immunological memory, and gnathostomes from shark to man were considered the sole possessors of “adaptive” immunity, the premise for preemptive vaccination against infectious disease.

**Figure 1:**
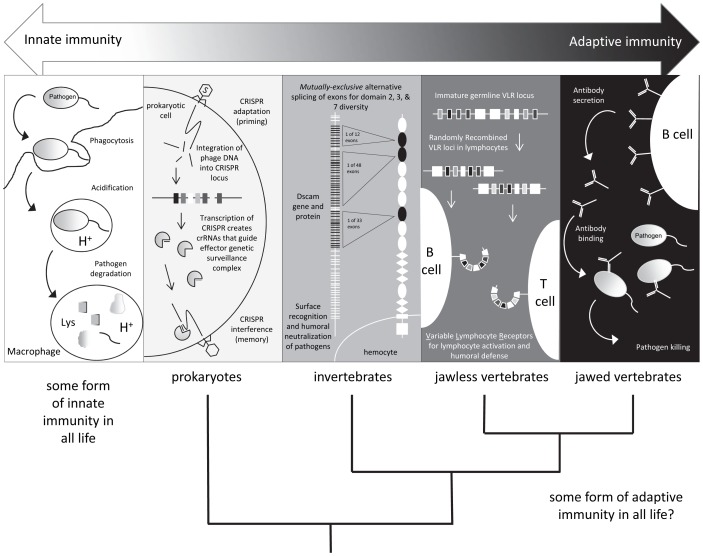
Shades of immunity. All life has innate immune mechanisms and jawed vertebrates have the IgSF lymphocyte receptor–based adaptive system as well. Different immune mechanisms with adaptive properties are being discovered in species originally considered to only possess innate immunity.

Recent studies in a wide range of species, however, have revealed unexpected forms of immune responses demonstrating specificity and immunological memory (Table 1). For example, recent studies in divergent arthropods have revealed that past exposure to a pathogen may provide individual organisms, or their descendants, with enhanced—and often times species-specific—protection against subsequent assaults. After an individual copepod *Macrocyclops albidus* was infected with a specific strain of the cestode parasite *Schistocephalus soliduse* early in life, the individual displayed enhanced and specific immunity against re-infection success and pathogen load [1]. In the waterflea *Daphnia magna*, individuals and their offspring exposed to a strain of their microparasite *Pasteuria ramosa* showed increased resistance against this specific strain as measured by fitness but not against a second tested strain [2], [3]. Finally, the bumblebee *Bombus terrestris* was shown to display pathogen species-specific immunity against two closely related taxa of the genus *Paenibacillus* [4] several weeks after immunization. The mechanisms underlying this prophylactic effect, termed immunological priming with vertebrate lymphocytes, remain an area of active investigation. However, some have speculated that synergistic interactions among components of the innate immune system could drive long-lasting changes in the immune responses of infected individuals [5]. Thus, the definition of adaptive immunity and the phylogenetic clades that possess it are not as clear as once thought: “shades of grey” have emerged (Figure 1).

**Table 1:** Examples of adaptive-like immune mechanisms outside of the jawed vertebrates.

Host	Finding	Reference
Copepod (*Macrocyclops*)	Success of challenge infection with tapeworm strain suggests immune specificity and memory	Kurtz and Franz, *Nature* 2003 [1]
Water flea (*Daphnia*)	Previous exposure confers protection from bacterial pathogen challenge and maternal transfer of protection	McTaggart et al., *Biology Letters* 2012 [2]; Little et al., *Current Biology* 2003 [3]
Bumblebee (*Bombus terrestris*)	Specific protection upon secondary exposure to congeneric bacterial pathogens	Sadd et al., *Current Biology* 2006 [4]
Lamprey (*Petromyzon marinus*)	VLRs rearrange humoral and cell-mediated (thymic) adaptive immune repertoires	Pancer *et al.*, *Nature* 2004 [7]; Guo et al., *Nature* 2009 [24]; Bajoghli et al., *Nature* 2011 [25]
Sea urchin (*Strongylocentrotus purpuratus*)	Explosion of hundreds of TLR/NLR gene variants	Rast et al., *Science* 2006 [11]
Dipteran flies (*Drosophila*, *Anopheles*)	Dscam mutually exclusive alternative splicing for large hemocyte receptor repertoire	Watson *et al.*, *Science* 2005 [13]; Dong *et al.*, *PLoS Biology* 2006 [14]
Plants (*Arabidosis thaliana*)	Plant *R* genes encode many genes that convey plant resistance to particular pathogens, often using leucine rich repeat domains (like TLR, VLR)	Jones and Dangl, *Nature* 2006 [26]
Snail (*Biomphalaria glabrata*)	Snail fibrinogen-related proteins (FREPs) are somatically diversified IgSF proteins that defend against schistosomes	Zhang et al., *Science* 2004 [27]
Sea urchin (*Strongylocentrotus purpuratus*)	Sp185/333 large family (∼50) of genes encode phagocyte receptors for bacteria that are diversified by unresolved mechanism	Smith, *Frontiers in Immunology* 2012 [28]
Tunicate (*Ciona intestinalis*)	Primitive chordates employ polygenic, highly polymorphic IgSF Variable Domain Chitin-binding Proteins (VCBPs) for mucosal immunity	Dishaw et al., *PNAS* 2011 [29]
Bacteria and archaea (*Escherichia coli*)	CRISPR loci capture viral DNA to direct future sequence-specific immune response against bacteriophages	Wiedenheft et al., *Nature* 2012 [16]; Barrangou et al., *Science* 2007 [17]

## (2) The Immunoglobulin Superfamily (IgSF) Is Neither the Only Nor the Oldest Antigen Receptor System

The immune cells of mammals employ complex families of immune receptors to respond to attacks by invading pathogens. The explosion of whole genome sequence information from phylogenetically diverse organisms has thrown into sharp relief the ways in which the adaptive immune receptors generally differ in quality and quantity from those of innate systems (Table 2). Immunoglobulins, T cell receptors (TCRs), and major histocompatibility complex molecules contain structural domains of the IGSF, but the IgSF is also used in molecules not involved in immunity.

**Table 2:** Canonical characteristics of immune receptors (adapted from Fig. 3.1 *Janeway's Immunobiology* 8th ed.) [30].

Innate	Adaptive
Specificity inherited in genome	Encoded in multiple gene segments
Expressed by all cells of a type	Requires somatic gene diversification
Ligation triggers immediate response	Clonal cellular distribution
Recognizes broad class of pathogen	Can discriminate between very similar structures
Binds range of structures of a given type	

In mammals and other gnathostomes, somatic genetic recombination of DNA provides the mechanism by which diversity is introduced into molecules that mediate immune recognition, including immunoglobulins and similar TCRs. In rearrangement catalyzed by the recombination-activating gene (RAG) products, which takes place in primary lymphoid tissue, variable, diverse, and joining immunoglobulin gene regions combine to generate libraries of immune molecules that possess the capacity to recognize diverse pathogen-associated antigens. After culling of self-reactive clones, interactions between diverse immunoglobulins and TCRs with antigens drive immune reactions and confer powerful, specific defense. However, the RAG locus evolved long before immunoglobulins [6], and RAG-based V(D)J diversification is not the only mechanism by which vast receptor repertoires, critical to combating specific pathogens, can be produced. The jawless agnathans (lampreys and hagfish) evolved for their lymphocytes an elaborate receptor system structurally more akin to toll-like receptors (TLRs) (using leucine-rich repeat domains) than immunoglobulins. These variable lymphocyte receptor (VLR) repertoires are formed by continual somatic diversification of a complex antigen receptor locus [7] prior to pathogen exposure and explain older phenomenology showing a memory response in these vertebrates where immunoglobulins could not be identified [8]. Hence, the elegant immunogenetic trickery of the RAG recombinase upon IgSF loci that arose in sharks and is enjoyed by man is (at least) the second system of lymphocyte antigen receptor diversification to evolve in vertebrates.

## (3) Invertebrate Immune Cells Have Complex Receptor Systems, Possibly Affording Adaptive Immunity

Innate immune receptors generally are neither clonally distributed nor encoded in a large number of genes or gene segments like their adaptive antigen receptor counterparts (Table 2). TLRs are evolutionarily conserved transmembrane proteins that play critical roles in mediating innate (and subsequently activating adaptive) immune defense in divergent taxa. TLRs are pattern recognition receptors (PRRs) that recognize specific pathogen-associated molecular patterns (PAMPs), molecules that are common to diverse classes of pathogens but significantly are not produced by the host. PAMPs recognized by TLRs include lipopolysaccharides, an abundant component of bacterial cell walls, and nucleic acid variants normally associated with viruses, including double-stranded RNA [9]. The human genome encodes 13 TLRs. However, whole genome sequencing of diverse species has revealed enormous variation in the number and diversity of these receptor genes. For example, amphioxus contains 71 [10] and the purple sea urchin genome contains genes encoding more than 200 [11] TLRs and other predicted PRRs that employ leucine-rich repeat domains (such as the NOD-like receptors, NLRs). The striking differences in the abundances of TLRs and NLRs hint that these molecules display divergent functions in disparate biological systems. It has been hypothesized that a need for enhanced specialization or recognition of PAMPs may contribute to expansion of the TLR gene family in these organisms [12]. Humans, on the other hand, possess a robust adaptive immune system and thus may have not needed to evolve TLR/NLR systems that are similarly elaborated. Additionally, the human TLR system is limited to immune recognition of threats exogenous and endogenous. Whereas, in other biological systems including the fruit fly, TLRs play roles in developmental processes. These observations suggest that expanded families of immune receptors may also comprise source material for the evolution of novel biological functions.

Just as the TLR family has evolved for use in nonimmune and immune roles, the Down syndrome cell adhesion molecule (Dscam) gene encodes a repertoire of receptors for axon-guidance in the human embryonic central nervous system, while its ortholog functions neuronally and also produces a gamut of immune receptors and secreted effectors of *Drosophila* fat body hemocytes. Dscam contains many IgSF domains, some of which are greatly diversified. Instead of somatic cell DNA rearrangement, as is typically found in vertebrate antibody genes, Dscam genes are subject to mutually exclusive alternative splicing, which results in large arrays of exons encoding more than ∼38,000 isoforms [13]. In the fruit fly, individual isoforms recognize bacteria differentially, whereas in the mosquito, silencing the Dscam ortholog weakens the resistance to infection by bacteria and the malaria parasite [14]. TLRs and Dscam are just two examples (Table 1) of large immune repertoires that operate in nonvertebrates, and determining the level of specificity of these receptors and the possibility of an anamnestic response constitute exciting avenues for future research.

## (4) Forms of Immunological Memory May Well Exist in Nonvertebrates, Even in Prokaryotes

Recently, novel mechanisms that mediate a form of adaptive immunity and immunological memory in bacteria have been described. Bacteria are assaulted by bacteriophages (viruses that infect bacteria), which can compromise or threaten host viability. To address this threat, bacteria have evolved diversity-generating retroelements, restriction enzyme modification, and phase variation mechanisms, which alter the susceptibility of bacteria to phage attachment, internalization, and attack [15]. However, these mechanisms do not confer species-specific immunological memory, and thus, a novel adaptive bacterial immunological system has evolved to provide an additional layer of immune protection in Bacteria and Archaea [16]. The clustered regularly interspaced short palindromic repeats (CRISPR) locus contains repetitive sequences interleaved with captured pathogen sequences that confer resistance to exogenous genetic elements by recognizing and degrading invading nucleic acids through a conserved catabolic process. Briefly, invading nucleic acids are integrated into the bacterial genome at the CRISPR locus, thereby maintaining a record of specific pathogens that have successfully invaded the pathogen. Importantly, CRISPR loci can be transcribed and processed into collections of short CRISPR-derived RNAs (crRNAs). These nucleic acids, in a manner analogous to RNAi in mammalian cells, can hybridize to invading nucleic acids and drive their destruction upon detection. The plastic CRISPR locus maintains immunogenetic memory of plasmids and phage and thus is under selective pressure itself to maintain the most useful repertoire. The locus has also evolved protective mechanisms preventing autoimmune attacks from the CRISPR RNA silencing effector mechanisms [17]. So in stark contrast to what many of us were taught as immunology schoolchildren, adaptive immunity (with its hallmark characteristics of specificity and memory) may be nearly as old as cellular life itself.

## (5) Comparative Immunologists Will Not Be the Sole Beneficiaries of These Discoveries

As more nontraditional model species are rigorously explored, unheralded domains, nucleic-acid-guiding systems, receptors, and immunogenetic diversification mechanisms will continue to surprise. The specificity required of adaptive immune systems naturally lends their components to many applications. The decades of understanding of antibodies and the loci that encode them have made their use common on our lab benches and in our clinics—even home pregnancy tests rely upon antibodies in an enzyme-linked immunosorbant assay. Similarly, now lamprey VLRs are being used as tools for selective recognition of glycans poorly discriminated by immunoglobulins [18]. The structure of a VLR binding the immunodominant glycoprotein of *Bacillus anthracis* has been solved, suggesting new diagnostic capabilities [19]. Dscam variable domains have been found to use a symmetrical, antiparallel, homophilic binding for self-process recognition that offers new avenues in protein barcoding for identification [20]. As we continue to understand and mine the diversity in immune mechanisms, we will find a wealth of macromolecular systems for exquisite molecular recognition, often with storage of that data cellularly or genetically. These systems have been battle-tested for millions of years and are ready for translation to our laboratory, clinical, industrial, and personal needs.

## Conclusion

The descriptive and phylogenetic demarcations of adaptive immunity may continue to require revision, and perhaps less concrete boundaries. As adaptive as some of the discussed nonvertebrate systems seem, clonal selection and tolerance thus far seem to be largely lacking or not understood, which may become important in new definitions. While “our” adaptive immunity surely evolved in a shark-like ancestor ∼460 million years ago [21]–[23] and has evolved tiers of regulation and complexity that should rightly dominate immunological research, we should also explore immunity in all life forms without preconceived notions of what we'll find. Are there other protein domains as often used for defensive repertoires as the IgSF and leucine-rich repeat? Have we just scratched the surface of nucleic acid/RNA-guided mechanisms to be discovered? What will epigenetic regulation add to our understanding? Clearly, natural selection has found many ways to defend self from non-self—many adaptive, many innate, and many shades that will require new categories in between.
